# A novel red fluorescence dopamine biosensor selectively detects dopamine in the presence of norepinephrine in vitro

**DOI:** 10.1186/s13041-021-00882-8

**Published:** 2021-12-06

**Authors:** Chihiro Nakamoto, Yuhei Goto, Yoko Tomizawa, Yuko Fukata, Masaki Fukata, Kasper Harpsøe, David E. Gloriam, Kazuhiro Aoki, Tomonori Takeuchi

**Affiliations:** 1 Department of Biomedicine, Aarhus University, Hoegh-Guldbergsgade 10, 8000 Aarhus C, Denmark; 2 Danish Research Institute of Translational Neuroscience - DANDRITE, Nordic-EMBL Partnership for Molecular Medicine, Aarhus University, Hoegh-Guldbergsgade 10, 8000 Aarhus C, Denmark; 3 Center for Proteins in Memory - PROMEMO, Danish National Research Foundation, Department of Biomedicine, Aarhus University, Hoegh-Guldbergsgade 10, 8000 Aarhus C, Denmark; 4 Quantitative Biology Research Group, Exploratory Research Center on Life and Living Systems (ExCELLS), National Institutes of Natural Sciences, 5-1 Higashiyama, Myodaiji-cho, Okazaki, Aichi 444-8787 Japan; 5 Division of Quantitative Biology, National Institute for Basic Biology, National Institutes of Natural Sciences, 5-1 Higashiyama, Myodaiji-cho, Okazaki, Aichi 444-8787 Japan; 6 Department of Basic Biology, School of Life Science, SOKENDAI (The Graduate University for Advanced Studies), 5-1 Higashiyama, Myodaiji-cho, Okazaki, Aichi 444-8787 Japan; 7 Division of Membrane Physiology, Department of Molecular and Cellular Physiology, National Institute for Physiological Sciences, National Institutes of Natural Sciences, 5-1 Higashiyama, Myodaiji-cho, Okazaki, Aichi 444-8787 Japan; 8 Department of Physiological Sciences, School of Life Science, SOKENDAI (The Graduate University for Advanced Studies), 5-1 Higashiyama, Myodaiji-cho, Okazaki, Aichi 444-8787 Japan; 9 Department of Drug Design and Pharmacology, University of Copenhagen, Universitetsparken 2, 2100 Copenhagen, Denmark

**Keywords:** GPCR, Dopamine, Norepinephrine, Fluorescence probe, Hippocampal neuron

## Abstract

Dopamine (DA) and norepinephrine (NE) are pivotal neuromodulators that regulate a broad range of brain functions, often in concert. Despite their physiological importance, untangling the relationship between DA and NE in the fine control of output function is currently challenging, primarily due to a lack of techniques to allow the observation of spatiotemporal dynamics with sufficiently high selectivity. Although genetically encoded fluorescent biosensors have been developed to detect DA, their poor selectivity prevents distinguishing DA from NE. Here, we report the development of a red fluorescent genetically encoded GPCR (G protein-coupled receptor)-activation reporter for DA termed ‘R-GenGAR-DA’. More specifically, a circular permutated red fluorescent protein (cpmApple) was replaced by the third intracellular loop of human DA receptor D1 (DRD1) followed by the screening of mutants within the linkers between DRD1 and cpmApple. We developed two variants: R-GenGAR-DA1.1, which brightened following DA stimulation, and R-GenGAR-DA1.2, which dimmed. R-GenGAR-DA1.2 demonstrated a reasonable dynamic range (*ΔF/F*_*0*_ = − 43%), DA affinity (EC_50_ = 0.92 µM) and high selectivity for DA over NE (66-fold) in HeLa cells. Taking advantage of the high selectivity of R-GenGAR-DA1.2, we monitored DA in presence of NE using dual-color fluorescence live imaging, combined with the green-NE biosensor GRAB_NE1m_, which has high selectivity for NE over DA (> 350-fold) in HeLa cells and hippocampal neurons grown from primary culture. Thus, this is a first step toward the multiplex imaging of these neurotransmitters in, for example, freely moving animals, which will provide new opportunities to advance our understanding of the high spatiotemporal dynamics of DA and NE in normal and abnormal brain function.

**Supplementary Information:**

The online version contains supplementary material available at 10.1186/s13041-021-00882-8.

## Introduction

The catecholaminergic neuromodulators dopamine (DA) and norepinephrine (NE) have very high structural similarity, differing only by a single hydroxy group. Dopaminergic projections mainly originate from the ventral tegmental area and the substantia nigra pars compacta [1], whilst noradrenergic projections mainly originate from the locus coeruleus (LC) [2, 3]. It was discovered recently that noradrenergic LC axons co-released DA along with NE [4–6]. DA is involved in reward [7–9], motivation [10], novelty response [11], and motor control [12, 13]. In addition, the involvement of DA and NE overlap in many brain functions [14, 15], such as learning and memory [11, 16], arousal [17, 18], and stress response [6, 19]. In particular, the prefrontal cortex receives both dopaminergic and noradrenergic projections, and these systems are involved in attention [20, 21] and working memory [22–24]. Furthermore, dysfunction of dopaminergic or noradrenergic systems are thought to be associated with psychiatric disorders and neurodegenerative diseases, such as attention-deficit/hyperactivity disorder (ADHD), schizophrenia, and Parkinson’s disease [25–27].

Interactions between DA and NE theoretically depend on the timing of release, spatial diffusions, concentrations, and DA/NE ratios. However, due to technical limitations, little is actually known about these properties with high spatial and temporal resolution within the same preparation. For example, microdialysis with high-performance liquid chromatography has high sensitivity and selectivity to detect either DA or NE but suffers from poor spatial and temporal resolution [28, 29]. In contrast, fast-scan cyclic voltammetry [30] and a synthetic catecholamine nanosensor [31] both have higher sensitivity and temporal resolution, but cannot distinguish between DA and NE. A method combining sensitivity, specificity, and spatiotemporal resolution is required to satisfactorily answer research questions regarding the timing of release, spatial diffusions, concentrations, and DA/NE ratios.

Recently developed genetically encoded fluorescent biosensors are able to detect extracellular DA or NE with high spatial and temporal resolution, and sensitivity in freely moving animals using in vivo imaging [32–34]. Binding of DA or NE to the sensor induces a conformational change, which couples with a change in the fluorescence of circular-permutated fluorescent protein, such as green fluorescent protein (GFP) for green fluorescence [32–34]. The green fluorescent NE (green-NE) biosensor, GRAB_NE1m_, has a high selectivity for NE (> 350-fold selectivity for NE over DA) [34]. However, current green fluorescent DA (green-DA) biosensors do not have high enough selectivity for DA over NE [32, 33], and they also have a high degree of spectral overlap with the green-NE biosensor. Consequently, it is difficult to use these green-DA biosensors for the simultaneous detection of DA and NE.

To image DA and NE dynamics simultaneously with high spatial and temporal resolution, we independently developed a circular-permutated mApple (cpmApple)-based red fluorescent DA (red-DA) biosensor, two other groups very recently reported red-DA biosensors, rGRAB_DA_ [35] and RdLight1 [36] though. We developed two variants: R-GenGAR-DA1.1, which brightened following DA stimulation, and R-GenGAR-DA1.2, which dimmed. R-GenGAR-DA1.2 demonstrated high selectivity for DA (66-fold selectivity for DA over NE) in HeLa cells. Taking advantage of the high selectivity of R-GenGAR-DA1.2, we monitored DA in presence of NE using dual-color fluorescence live imaging, combined with the green-NE biosensor GRAB_NE1m_ [34] in HeLa cells and in hippocampal neurons grown from primary culture.

## Methods

### Animals

This study was not pre-registered. No blinding, randomization or sample size calculations were performed. Inclusion/exclusion criteria were not used in this study. Animal experiments were approved by the Animal Care Committee of the National Institutes of Natural Sciences in Japan (19A029) and were performed in accordance with its guidelines.

### Compounds used to test biosensor fluorescence response

Dopamine (DA) hydrochloride (1 M stock, H8602, Sigma-Aldrich), serotonin hydrochloride (50 mM stock, 14332, CAY), and L-adrenaline (epinephrine) (5 mM stock, A0173, TCI) were dissolved in 10 mM HCl. L-noradrenaline bitartrate monohydrate (1 M stock, A0906, TCI), sodium L-glutamate monohydrate (10 mM stock, G0188, TCI), 4-aminobutyric acid (100 mM stock, A0282, TCI), histamine (100 mM stock, 18111–71, Nacalai Tesque), acetylcholine chloride (10 mM stock, A6625, Sigma-Aldrich), R( +)-SCH 23390 hydrochloride (10 mM stock, D054, Sigma-Aldrich), and octopamine hydrochloride (10 mM stock, O0413, TCI) were each dissolved separately in distilled water. SKF 81297 hydrobromide (10 mM stock, 1447, TOCRIS), haloperidol hydrochloride (20 mM stock, 0931, TOCRIS), yohimbine hydrochloride (20 mM stock, 1127, TOCRIS), and tyramine (10 mM stock, A0302, TCI) were dissolved in DMSO. Compound solutions were then subdivided into aliquots and stored at − 20 °C until use. A working solution of 1 M DA was stored at 4 °C for 3 weeks prior to use.

### Plasmids

**Fig. 1:**
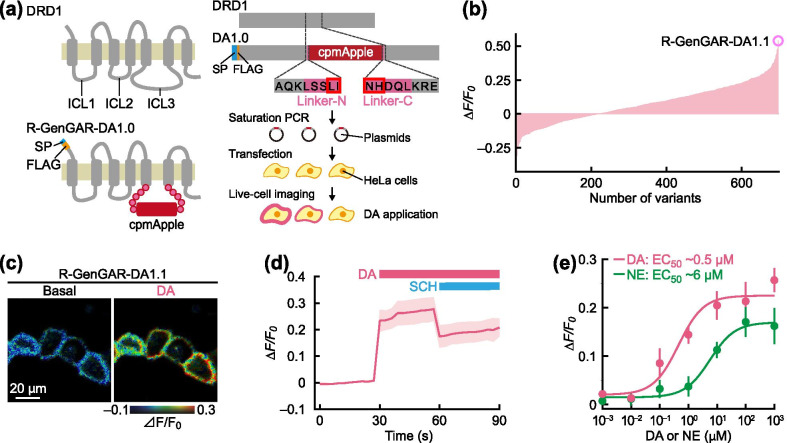
Development of R-GenGAR-DA1.1, which showed a positive response to dopamine (DA). **a** Strategy to develop R-GenGAR-DA1.0. Left panels show a schematic illustration of human DRD1 and the red fluorescent protein ‘cpmApple’ insertion site. Right panels show screening flow chart. Linker sequences connecting DRD1 and cpmApple were randomly mutated using saturation PCR. Mutation sites in the linker are shown in red boxes. The plasmids expressing each linker mutant were isolated, followed by the transfection into HeLa cells by lipofection. Changes in fluorescence intensity following 10 μM DA stimulation was monitored by live-cell imaging of HeLa cells expressing each mutant. ICL, intracellular loop; SP, signal peptide (hemagglutinin secretory sequence). **b** Summary of screening results. The fluorescence changes (*ΔF/F*_*0*_) of the HeLa cells expressing each mutant in response to 10 μM DA stimulation are shown. Each bar represents the average of 1–3 independent experiments. We selected a mutant “R-GenGAR-DA1.1” that showed a maximum response to 10 μM DA stimulation. **c** Representative images of HeLa cells expressing DA1.1 stimulated with 10 µM DA. The fluorescence change (*ΔF/F*_*0*_) before and after DA stimulation are shown in the pseudocolor intensity-modulated display mode. **d** The fluorescence change (*ΔF/F*_*0*_) of DA1.1 in HeLa cells in panel (**c**). DA (10 µM) and SCH 23,390 (SCH, 10 µM) were treated at the time points indicated by pink and blue bars, respectively (Additional file 1: Fig. S5a). Mean *ΔF/F*_*0*_ values of 10 cells from 1 experiment are shown with SD (shaded area). **e** Dose–response curves, with temperature equilibration (Additional file 1: Fig. S4d), of DA (pink) and NE (green) in HeLa cells expressing DA1.1. DA: max *ΔF/F*_*0*_ = 0.23 ± 0.02 and EC_50_ = 0.45 ± 0.21 µM; NE: max *ΔF/F*_*0*_ = 0.17 ± 0.03 and EC_50_ = 5.68 ± 1.19 µM (DA and NE, *n* = 3 experiments in both cases, 10 cells per experiment). Experimental data (dots) were fitted with the Hill equation (lines). DA1.1 has 12.6-fold selectivity for DA over NE

R-GenGAR-DA1.0 cDNA and dLight1.1 [32] and RdLight1 [36] cDNA were synthesized by FASMAC into the vector plasmid pUCFa (FASMAC). We used a cpmApple module with linker sequences (LSS-LI-cpmApple-NH-DQL) from RGECO1 [37], which was a gift from Dr. Takeharu Nagai, for insertion into human DRD1 (NP_000785). Sequences coding for hemagglutinin (HA) secretion motif and a FLAG epitope were placed at the 5’ end of the construct as in dLight1.1 [32] (Fig. 1a). *Eco*RI and *Not*I recognition sites were placed at the 5’ and 3’ end, respectively, for subcloning into the expression vector, pCAGGS [38] with ligation using Ligation High ver.2 (LGK-201, TOYOBO). Point mutations of R-GenGAR-DA1.0, and dLight1.2 and dLight1.3a [32] were made using polymerase chain reaction (PCR) with the primers containing each mutation and PCR enzyme mixture KOD One (KMM-201, TOYOBO). GRAB_NE1m_ [34] was provided by Dr. Yulong Li and subcloned into the pCAGGS. rGRAB_DA1h_ (#140557, Addgene) and rGRAB_DA1m_ (#140556, Addgene) were purchased and subcloned into the pCAGGS.

### Saturation PCR for the screening of optimal linker sequences

To maximize the fluorescence changes according to the conformational change of R-GenGAR-DA1.0, optimized linker sequences were screened by the saturated PCR. Primers with random bases encoding two-amino acid length were designed as follows. Forward Primer: 5’-TTGCTCAGAAACTTTCAAGTNNBNNBGTGTCCGAAAGAATGTACCC-3’; Reverse Primer: 5’-GTTTCTCTTTTCAACTGATCVNNVNNTGCCTCCCACCCCATAGTTT-3’.

Randomized linker sequences and cpmApple were amplified by PCR and inserted into pUCFa-DRD1-cpmApple plasmid with NEBuilder HiFi DNA assembly (E2621, NEB). These mutant plasmids were transformed into *E.coli* and purified as library plasmids. The library plasmids were digested by *Eco*RI and *Not*I to extract library insert. Library inserts were subcloned into the pCAGGS vector by ligation and transformation into *E.coli*. Single *E.coli* colonies were picked up and the plasmids were prepared from them. Each plasmid was transfected into HeLa cells (see details below) seeded in 96-well glass-bottom plate with 293-fectin (12347019, Thermo Fisher Scientific). Two days after the transfection, cells were imaged as described below.

### Design of DRD1 mutations based on structural models

Structural models of DRD1 with DA and NE were constructed using PyMOL (The PyMOL Molecular Graphics System, Version 2.0 Schrödinger, LLC) from a crystal structure of the related β_2_-adrenoceptor with bound epinephrine [39] downloaded from the RCSB Protein Data Bank web site (http://www.pdb.org; PDB code, 4LDO). The binding site residues with side-chain atoms within 5 Å of epinephrine’s aliphatic hydroxy group was exchanged for those of DRD1 by selecting high-probability backbone-dependent rotamers suggested by the mutagenesis wizard in PyMOL. DA was built by deleting the additional methyl and the aliphatic hydroxy groups and NE by deleting only the methyl group. Using the same cut-off as above, Ser 107, Val 317 and Trp 321 were identified as residues that could potentially interact with the extra hydroxy group on NE. Asp 103 was disregarded as it is essential for binding of both agonists by interacting with the protonated amine.

With the aim of lowering the binding affinity of NE by removing a potential hydrogen bond to the aliphatic hydroxy of NE, Ser 107 was mutated to Cys and Ala. Additionally, to introduce steric hinderance around the aliphatic hydroxy group, Ile, Leu, Met and Val mutations were also performed. Val 317 was mutated to other hydrophobic residues with longer side chains (Ile, Leu, Phe and Met), again to introduce steric hinderance around the hydroxy in NE. Trp 321 was first mutated to Phe to remove the hydrogen bonding possibility whilst maintaining aromaticity, but since this was detrimental to DA and NE binding, we attempted other residues that maintained hydrogen bonding possibility (His and Gln).

### Cell culture

We used the HeLa cell line (provided by Michiyuki Matsuda). HeLa cells were cultured in DMEM (08459-64, Nacalai Tesque) supplemented with 10% fetal bovine serum (F7524, Sigma-Aldrich) at 37 °C in 5% CO_2_. HeLa cells were cultured up to passage 30. We have not authenticated this HeLa cell line since receipt. However, the HeLa cell line is not listed as a common misidentified cell line by the ICLAC (https://iclac.org/databases/cross-contaminations/). HeLa cells (3 × 10^4^ cells/well) were plated on CELLview cell culture dishes (glass bottom, 35 mm diameter, 4 compartments, 62787, Greiner Bio-One) (Additional file 1: Fig. S4a) one day before transfection. Transfection was performed by incubating the cells with a mixture containing 250 ng DNA and 0.25 µl 293fectin transfection reagent (12347019, Thermo Fisher Scientific) per well for 4–6 h. Imaging was performed 2 days after transfection.

Primary cultures of rat hippocampal neurons were prepared similarly to that described previously [40]. Female pregnant Wistar/ST rats (total 8 rats) arrived from Japan SLC on the day of the experiment. A pregnant rat with embryonic rats (embryonic days 19) was killed by CO_2_ inhalation and then embryos (10 embryos per pregnant rat) were removed and decapitated. Hippocampi were dissected from embryonic rat brains and placed in a 10-cm dish on ice with a Hanks’ buffered saline solution (17461-05, nacalai tesque) with 10 mM glucose and 10 mM HEPES (pH 7.4) (H0887, Sigma-Aldrich). To dissociate hippocampal neurons, hippocampi were treated with 10 units/ml papain (LS003126, Worthington Biochemical) for 10 min at 37 °C. Dissociated neurons were plated onto poly-L-lysine (P6282, Sigma-Aldrich)-coated 35 mm-glass-bottom dishes (3 × 10^5^ cells/well) (Additional file 1: Fig. S4a) with a plating medium containing: neurobasal medium (21103-049, Thermo Fisher Scientific), 10% fetal bovine serum (172012, Sigma-Aldrich) and 10 mM HEPES (pH 7.4). Neurons were incubated at 37 °C and 5% CO_2_ for 3 h, and then the medium was replaced with neurobasal medium with B-27 supplement (17504044, Thermo Fisher Scientific), 2 mM GlutaMax supplement-I (35050061, Thermo Fisher Scientific), and 10 mM HEPES (pH 7.4). Half of the medium was removed and replaced with fresh medium every 7 days. The cultured neurons were transfected at 14–21 days in vitro by Lipofectamine 2000 (11668019, Thermo Fisher Scientific) and were imaged 4–6 days after transfection.

### Fluorescence imaging

For the imaging of HeLa cells, the medium was changed to imaging buffer [FluoroBrite-DMEM (FB), A1896701, Life Technologies] supplemented with 1% GlutaMax (35050061, Thermo Fisher Scientific), and 0.2% fetal bovine serum (F7524, Sigma-Aldrich) at least 2 h before imaging. For primary hippocampal neurons, the medium was changed to HBSS [119 mM NaCl, 5 mM KCl, 2 mM CaCl_2_, 25 mM HEPES (pH 7.4), 2 mM MgCl_2_, and 33 mM D-glucose] before imaging started.

For the screening of optimal linkers, HeLa cells transfected with library plasmids were imaged with a high content imaging system, IXM-XLS (Molecular Device), equipped with an air objective lens (CFI Plan Fluor 10 × , NA = 0.30, WD = 16 mm and CFI Plan Apochromat Lambda 20 × , NA = 0.75, WD = 1 mm; Nikon), a Zyla 5.5 sCMOS camera (ANDOR), and a SOLA SE II light source (Lumencor). The excitation and fluorescence filter settings were as follows: excitation filter 562/40 (FF01-562/40–25), dichroic mirror 350–585/601–950 (T) (FF593-Di03-25 × 36), and emission fluorescence filter 624/40 (FF01-624/40–25) purchased from Semrock. Fluorescence changes before and after application of 10 μM DA were imaged by the IXM-XLS (Molecular Device).

Confocal fluorescence imaging of cells were imaged with an IX83 inverted microscope (Olympus) equipped with a sCMOS camera (Prime, Photometrics), an air objective lens (UPLSAPO 20 × , NA = 0.75, WD = 0.6 mm or UPLXAPO 20 × , NA = 0.8, WD = 0.6 mm; Olympus), an oil objective lens (UPLSAPO 60 × , NA = 1.35, WD = 0.15 mm or UPLXAPO 60 × , NA = 1.42, WD = 0.15 mm; Olympus) and a spinning disk confocal unit (CSU-W1, Yokogawa Electric Corporation), illuminated with a laser merge module containing 440-nm, 488-nm, and 561-nm lasers. The excitation laser and fluorescence filter settings were as follows: excitation laser, 440 nm [for cyan fluorescent protein (CFP) and fluorescence resonance energy transfer (FRET) with cyclic adenosine monophosphate (cAMP) biosensor)], 488 nm (for NE1m) and 561 nm (for DA1.2); excitation dichroic mirror, DM445/514/640 [for cyclic adenosine monophosphate (cAMP) biosensor; Yokogawa Electric], DM405/488/561 (for NE1m and DA1.2; Yokogawa Electric); emission filters 465–500 nm (CFP for cAMP biosensor; Yokogawa Electric), 500–550 nm (for NE1m and FRET for cAMP biosensor; Yokogawa Electric), and 580–654 nm (for DA1.2; Yokogawa Electric).

For measuring excitation and emission spectra, we used the lambda-scan function on the SP8 FALCON (Leica microsystems) equipped with a HC PL APO 20 × /0.75 CS2 correction ring (Leica microsystems). For the emission spectrum, excitation wavelength was fixed at 540 nm and a 10-nm emission window was used to scan the emission intensity from 550 to 697 nm. For the excitation spectrum, emission channel was fixed at 550 nm to 780 nm and excitation laser wavelength was scanned from 470 to 614 nm.

For measuring relative brightness, equal amounts of pCAGGS-EGFP were co-transfected into HeLa cells with pCAGGS-R-GenGAR-DA-1.2, RdLight1, rGRAB_DA1m_, and rGRAB_DA1h_. The fluorescent intensity of each red dopamine sensor was measured and this was then divided by the intensity of EGFP to normalize the expression. The signal-to-noise ratio was calculated as the response (*ΔF/F*_*0*_) divided by the standard deviation of the fluorescence fluctuation.

For the measurement of on- and off-rate, HeLa cells expressing R-GenGAR-DA1.2 were observed with the IX83 inverted microscope (Olympus) equipped with the spinning disk confocal unit (CSU-W1; Yokogawa Electric) and the oil objective lens (UPLXAPO 60 × , Olympus) in streaming mode. The sampling interval was 20 ms with the 5% 561 nm laser. Dopamine (10 μM) was applied at approximately 5 s following the start of observation for on-rate measurement, while SCH23390 (20 μM) was applied to DA pre-treated cells for off-rate measurement. Time series data were fit by a single exponential decay curve followed by the extraction of half time from the fitting curve.

All imaging conditions are summarized in Additional file 1 Table S1.

### Application of compounds for imaging

Stock solutions for the compounds were dissolved in the appropriate vehicle, and a volume from 0.95 to 1 µl was prepared in each 1.5-ml microcentrifuge tube. Compounds were mixed with 0.5 ml imaging buffer from the well and applied to the same well at each time point during imaging (Additional file 1: Fig. S4b). For temperature equilibration of the imaging buffer, 0.5 ml of the imaging buffer was transferred from the well into an empty 1.5-ml microcentrifuge tube and then applied to the buffer in the same well; this procedure repeated 5 times (Additional file 1: Fig. S4c). The procedure for the compound application in the time-lapse imaging is shown in Additional file 1: Fig. S5. The ‘ligand’ dissolved in the appropriate vehicle was applied at the imaging time point shown by the arrow; the ‘vehicle’ was applied at the same time point. The ‘control’ only had light exposure for evaluating the effects of fluorescence change induced by excitation light.

### Detection of cAMP signaling using a cAMP biosensor

The cAMP biosensor, which was developed based on previous work [41], contains monomeric teal fluorescent protein (mTFP), a monomeric cyan fluorescent protein (CFP), the human RAPGEF3 (EPAC) gene (corresponding to 149–881 a.a.) obtained from HeLa cells with RT-PCR, and mVenus, a monomeric yellow fluorescent protein (YFP). The cDNA of cAMP biosensor was inserted into a pCX4neo vector [42]. The plasmid was co-transfected with either DRD1-Tango, which was a gift from Dr. Bryan Roth (Addgene kit #1000000068) [43], DA1.2, or empty vector. The cells were imaged 2 days after transfection. The level of cAMP was calculated by the ratio of CFP to FRET, followed with normalization by the baseline value before DA application.

### Quantification of imaging and data analysis

We used Fiji, a distribution of ImageJ [44], for the preparation of quantification and measurement of all imaging files. Principally, for all images, the background was subtracted, and images were registered by StackReg (http://bigwww.epfl.ch/thevenaz/stackreg/), a Fiji plugin to correct misregistration, if required. Note that the median filter was used for the time-lapse images of the neuron before registration to remove camera noise preventing registration. Then, regions of interests (ROIs) were selected for the first time point in time-lapse imaging or in the images before the compound application, to surround the whole cell body for HeLa cells and a dendrite near the cell body for hippocampal neurons. Mean pixel intensity in ROIs were measured and these data were further analyzed using Python3 (https://www.python.org). In order to normalize the fluorescence changes with the amount of biosensor expression, *ΔF/F*_*0*_ was calculated using the intensity before the compound application. For the time-lapse experiments, the value used for *F*_*0*_ was the mean intensity before compound application. The image of fluorescence change (*ΔF/F*_*0*_) was represented as the pseudocolor intensity-modulated display mode, where color represents the relative ratio value, whilst the brightness of the color represented the fluorescence intensity of the source images.

The EC_50_ and the max *ΔF/F*_*0*_ values were obtained as follows: First, HeLa cells transiently expressing DA sensors were prepared, set on a microscope, and stimulated with DA or NE in ascending order of concentration. Only one field of view was imaged, and therefore, in the case of HeLa cells, dose-responses of about 10 cells were obtained from one field of view. The averaged dose–response from 10 cells was fitted with the Hill function, and the EC_50_ and the max *ΔF/F*_*0*_ values were obtained using the Python package Scipy1.4 (SciPy.org). Note that the Hill coefficient was fixed as 1 because no cooperative binding was expected. The EC_50_ and the max *ΔF/F*_*0*_ from multiple trials were averaged. In the case of hippocampal primary cultured neurons, only data from one cell could be obtained from one field of view due to relatively low gene transfection efficiency. The dose–response in each neuron was fitted with the Hill equation, providing the EC_50_ and the max *ΔF/F*_*0*_ values. These values were averaged among neurons.

### Statistical analysis

All data were presented as mean, with error bars indicating ± SEM if not otherwise specified. Statistical analyses were performed using Prism8 (GraphPad Software) and Python 3.0 (Python Software Foundation) with SciPy (SciPy.org) and scikit-posthocs (https://scikit-posthocs.readthedocs.io/) packages. Data were analyzed using Mann–Whitney *U*-test, Student’s *t*-test, one-way ANOVA followed by Dunnett’s or Tukey–Kramer’s post hoc tests as appropriate to correct for multiple comparisons, and the Friedman test followed by Conover-Iman test with the Bonferroni-Holm correction to correct for multiple comparisons. In Additional file 1: Fig. S3e, f, normality assumption was judged from Shapiro–Wilk test and Q-Q plot and variances among conditions was estimated to be equal using Bartlett test. We did not conduct tests for outliers and did not exclude data points. All statistical tests were two-tailed. The level of significance was set at *p* < 0.05.

## Results

### Development and characterization of a red fluorescence DA biosensor

To develop a genetically encoded red fluorescence DA (red-DA) biosensor, we adopted the same approach as used to develop the green-DA biosensors dLight [32] and GRAB_DA_ [33]. First, we constructed an initial red-DA biosensor variant by inserting a red fluorescent protein, cpmApple [37], with linker sequences between Lys 223 and Lys 265 of human DA receptor D1 (DRD1), similarly to that done to construct dLight. We named it red fluorescent genetically encoded GPCR activation reporter for DA, ‘R-GenGAR-DA1.0’ (abbreviated DA1.0; Fig. 1a). However, when DA was applied, DA1.0 did not exhibit a fluorescence response (Additional file 1: Fig. S1a). To improve its fluorescence response to DA, random mutagenesis was performed on the linker peptide sequences between DRD1 and cpmApple on DA1.0 (Fig. 1a). HeLa cells expressing mutants of DA1.0 were stimulated by application of DA, and the change in red fluorescence intensity was quantified (Fig. 1b). Of 864 mutants, we selected three mutants (#76, #310, and #430) that responded positively to DA and subjected them to time-lapse imaging (Fig. 1c, d, and Additional file 1: Fig. S1b). All three mutants showed detectable red fluorescence increases in response to DA application and this response was blocked by the DRD1/5 antagonist SCH 23390 (SCH) (Fig. 1c, d, and Additional file 1: Fig. S1b). The amino acid sequences of mutated linkers were determined in these mutants (Additional file 1: Fig. S1c). We selected #76 (‘R-GenGAR-DA1.1’, abbreviated DA1.1) because it showed the largest positive response to DA amongst the three mutants. We then characterized the dose–response curves of DA1.1 for DA and NE and calculated the half maximal effective concentration (EC_50_). As a result, DA1.1 showed 13-fold selectivity for DA over NE (Fig. 1e).

### Development and characterization of an inverse-type red-DA biosensor

**Fig. 2:**
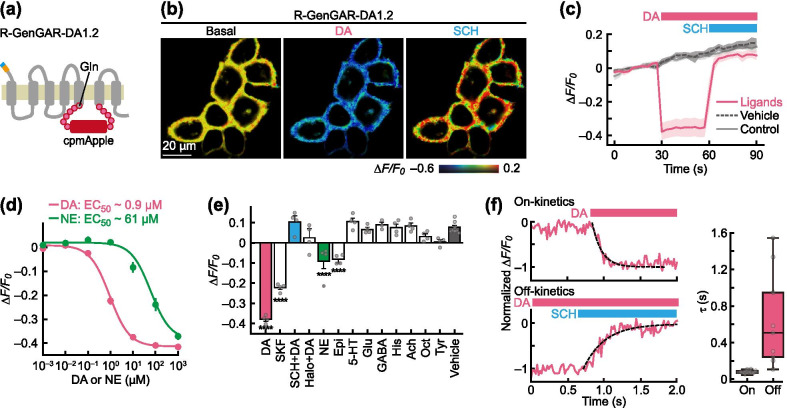
Development of R-GenGAR-DA1.2, which showed a negative red fluorescence response to DA. **a** Schematic illustration of a mutation site. Glutamine (Gln) was introduced into the N-terminal side of a linker in DA1.1. **b** Representative images of HeLa cells expressing DA1.2 treated with 10 µM DA, followed by 10 µM DRD1/5 antagonist SCH 23390 (SCH). Images are shown in the pseudocolor intensity-modulated display mode. **c** The fluorescence change (*ΔF/F*_*0*_) of DA1.2 in HeLa cells. Medium temperature was equilibrated before imaging (Additional file 1: Fig. S5b). DA (10 µM) and SCH (10 µM) were treated at time points indicated by pink and blue bars, respectively. Mean *ΔF/F*_*0*_ of 30 cells from 3 experiment are shown with SD (shaded area). Vehicle, 10 µM HCl or water; control, cells were only exposed to excitation light. **d** The dose–response curves, with temperature equilibration (Additional file 1: Fig. S4d), of DA (pink) and NE (green) in HeLa cells expressing DA1.2. DA: max *ΔF/F*_*0*_ = − 0.43 ± 0.01 and EC_50_ = 0.92 ± 0.13 µM; NE: max *ΔF/F*_*0*_ = − 0.43 ± 0.02 and EC_50_ = 61 ± 23 µM (DA, *n* = 8 experiments; NE, *n* = 10 experiments). Experimental data (dots) were fitted with the Hill equation (lines). DA1.2 has 66-fold selectivity for DA over NE. **e** Selectivity of DA1.2 for pharmacological compounds (*n* = 3–4 experiments, 10 cells per experiment; Additional file 1: Fig. S5c). All compounds = 10 µM. SKF 81297 (SKF), DRD1 agonist; haloperidol (Halo), DRD2 antagonist; Epi, epinephrine; 5-HT, serotonin; Glu, glutamate; GABA, γ-aminobutyric acid; His, histamine; Ach, acetylcholine; Oct, octopamine; Tyr, tyramine. For the vehicle condition, there was no significant difference between 10 µM HCl in H_2_O and 0.1% dimethyl sulphoxide (DMSO) (*n* = 4 experiments in each, 10 cells per experiment; Mann–Whitney *U*-test, *p* = 0.69). Therefore, these values were averaged and used as the vehicle condition. Mean *ΔF/F*_*0*_ values are shown with SEM. One-way ANOVA, *F*_12,38_ = 53.11, *p* < 0.0001; Dunnett's post hoc test (vs vehicle), ****p < 0.0001. **f** The on- and off-rate of DA1.2. The fluorescence intensity in each on- and off-kinetics was normalized by setting the maximum value as 0 and the minimum value as –1. The left panel indicates the time course of the normalized *ΔF/F*_*0*_ of DA1.2 (pink line) with the fitting curve (black dotted line). In the right panel, the on- and off-rates are shown as a box plot, in which the box shows the quartiles of data with the whiskers denoting the minimum and maximum, except for the outliers detected by 1.5 times the interquartile range (on-rate: 77 ± 5 ms, n = 13 cells; off-rate: 640 ± 160 ms, *n* = 9 cells)

The dynamic range of DA1.1 and its selectivity for DA were lower than some other DA biosensors [32, 33], prompting us to make further improvements. We attempted to expand the dynamic range of DA1.1 by introducing the same mutations as in the green-DA biosensor dLight1 [32]. Substitution of Phe 129 with Ala (F129A mutation of dLight1.2; Additional file 1: Fig. S2a), and addition of Gln to the N-terminal linker (dLight1.3a; Fig. 2a) were previously shown to significantly increase the dynamic range of dLight1.1 [32]. The F129A mutation in DA1.1, however, led to only a slight increase in the fluorescent signal upon DA application (Additional file 1: Fig. S2b) and its sensitivity to both DA and NE was lower than that of the original DA1.1 (data not shown). Surprisingly, the addition of Gln to the N-terminal linker in DA1.1 showed bright red fluorescence in the basal state. This variant had substantially reduced fluorescence signal in response to DA, which subsequently returned to basal level following treatment with SCH (Fig. 2b). We named this inverse type red-DA biosensor ‘R-GenGAR-DA1.2’ (abbreviated DA1.2).

Unexpectedly, time-lapse imaging of DA1.2 in HeLa cells showed that the baseline fluorescent signals increased gradually in both vehicle and control conditions (data not shown). We explored the cause of this phenomenon and found that the DA1.2 baseline fluorescent signals were associated with temperature-dependent fluorescence change, which is known as thermochromism [45] (Additional file 1: Fig. S3a–d). This effect was apparent from the inverse relationship between the baseline fluorescent signals of R-GenGAR-DA and incubation temperature (Additional file 1: Fig. S3a–d). Temperature-dependent fluorescence changes were observed when test compounds were added to our experimental system. Temperature equilibration of the medium minimized the effects of temperature-dependent change in fluorescence by making the temperature of the diluted compound the same as the temperature of the culture medium. Therefore, imaging was performed after temperature equilibration (Additional file 1: Figs. S4 and S5). However, we still observed an increase in basal red fluorescence of DA1.2 expressed in HeLa cells under constant medium temperature following application of the excitation light at a wavelength of either 561 nm, or both 488 nm and 561 nm, for repeated short-term intervals (Additional file 1: Fig. S3e). We also found that baseline fluorescence changes in DA1.2 induced by the excitation light were dependent on the intensity of light (Additional file 1: Fig. S3e). Furthermore, this increased baseline fluorescence change in DA1.2 was more pronounced in the imaging condition of primary hippocampal neurons (Additional file 1: Fig. S3f). We believe that baseline fluorescence changes induced by excitation light are indicative of photochromism [45]. We sought an optimal imaging condition and eventually found that the baseline fluorescence change induced by the excitation light reached a steady state after 150 s in our imaging condition in a primary culture of hippocampal neurons (Additional file 1: Fig. S3f). Pre-illumination minimized the effect of baseline fluorescence changes induced by excitation light by pre-saturating the proportion of fluorescent proteins that can be fluoresced by excitation light. Therefore, we performed pre-illumination to stabilize the baseline when the increased baseline fluorescence change was remarkable (Additional file 1: Fig. S5d and Additional file 1: Table S1).

Time-lapse imaging of DA1.2 with temperature equilibration (Additional file 1: Fig. S5b) showed that DA application lowered red fluorescence, which was restored following SCH treatment (Fig. 2c). Baseline fluorescence intensity still increased moderately in both vehicle and control conditions, possibly due to the baseline fluorescence change induced by excitation light. The dose–response curve with temperature equilibration (Additional file 1: Fig. S4d–f) shows that DA1.2 has a slightly higher dynamic range (1.7 times) and comparable affinity to DA (max *ΔF/F*_*0*_ = − 0.43 ± 0.01 and EC_50_ = 0.92 ± 0.13 μM) compared to that of DA1.1 (Fig. 2d). It is of note that the selectivity of DA1.2 for DA over NE was 66-fold, much higher than that of DA1.1 (Fig. 2d), which was due to a decrease in the affinity of DA1.2 to NE.

In order to further enhance the selectivity of DA1.2 for DA over NE, we attempted to predict mutations based on ligand-receptor structure models (see Methods). Since the difference between DA and NE is only one additional hydroxy group on NE, preference for DA might be accomplished by making the area around the binding site unfavorable for this hydroxy group. Based on a structural model complex of the DRD1 with either DA or NE in the binding site (Additional file 1: Fig. S6a), we then introduced 13 mutations to DA1.2 designed to increase preference for DA over NE (Additional file 1: Fig. S6b). Six of the 13 mutants showed a change in red fluorescence response to DA (Additional file 1: Fig. S6c, d). Although the dose–response curves with temperature equilibration for both DA and NE were obtained from three mutants, none of these demonstrated an increase in selectivity for DA over NE compared to that of DA1.2 (Additional file 1: Fig. S6e).

We then confirmed that the selectivity of the red-DA biosensor DA1.2 (66-fold) was higher than that of green-DA biosensors dLight1.1 (17-fold), dLight1.2 (32-fold), and dLight1.3a (19-fold) in our experimental conditions (Additional file 1: Fig. S7). We further compared the selectivity for DA over NE of DA1.2 to recently published red-DA biosensors [35, 36] and found that the selectivity of DA1.2 (66-fold) was higher than rGRAB_DA1h_ (1.6-fold), rGRAB_DA1m_ (8.5-fold), and RdLight1 (25-fold) (Additional file 1: Fig. S7).

Next, we characterized how DA1.2 responded to a variety of test compounds. The DRD1/5 agonist SKF 81297 led to a partial response from DA1.2, whilst the response to DA was blocked by the DRD1/5 antagonist SCH (Fig. 2e). The application of several other neurotransmitters/neuromodulators showed no significant response in DA1.2 (Fig. 2e). We further measured several more DA1.2 parameters, such as the on-rate (77 ± 5 ms) and off-rate (640 ± 160 ms) (Fig. 2f), the excitation and emission spectra (Additional file 1: Fig. S8a), relative brightness (Additional file 1: Fig. S8b), blue-light induced photoactivation (Additional file 1: Fig. S8c), and signal-to-noise ratio (Additional file 1: Fig. S8d). In addition, we confirmed that DA1.2 induced no cAMP increase upon DA application, indicating that, unlike wild-type DRD1, DA1.2 activity does not activate the canonical G_s_ signaling pathway (Additional file 1: Fig. S9).

### Dual-color fluorescence imaging of DA and NE in HeLa cells

**Fig. 3:**
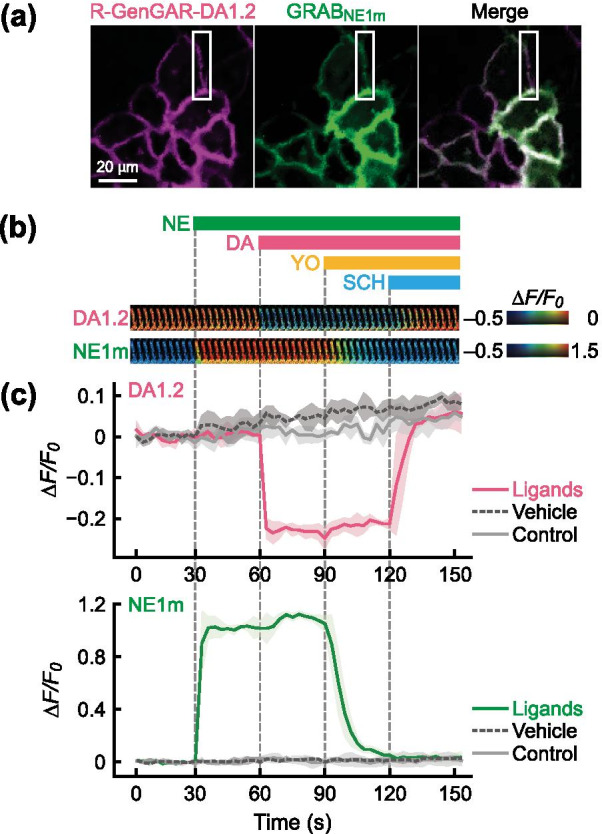
Dual-color fluorescence time-lapse imaging of the red-DA biosensor R-GenGAR-DA1.2 combined with the green-NE biosensor GRAB_NE1m_ in HeLa cells. **a** Representative image of HeLa cells co-expressing DA1.2 and NE1m. **b** Top: bars show the schedule of agonist/antagonist application to both DA1.2 and NE1m. Gray vertical lines indicate time of application. Concentrations: DA and SCH, 5 µM; NE and YO, 1 µM. Temperature equilibration and pre-illumination were conducted before compounds application (Additional file 1: Fig. S5d). Bottom: enlarged time-lapse images in the pseudocolor intensity-modulated display mode from the white boxed region shown in panel (**a**). **c** The fluorescence intensity change (*ΔF/F*_*0*_) of DA1.2 (top) and NE1m (bottom) in HeLa cells co-expressing DA1.2 and NE1m. Vehicle, 10 µM HCl in H_2_O or 0.1% DMSO; control, cells were only exposed to excitation light. Mean *ΔF/F*_*0*_ values of 30 cells from 3 experiments are shown with SD (shaded areas). Result of statistical test is shown in Additional file 1: Fig. S11a, b

We then tested the simultaneous imaging of DA and NE at the single-cell level. To accomplish this, both red-DA biosensor DA1.2, and a green-NE biosensor GRAB_NE1m_ (abbreviated NE1m) which has high selectivity for NE over DA (> 350-fold) [34], were co-expressed in HeLa cells (Fig. 3a). Because excitation light induced a change in baseline fluorescence during dual-color imaging of DA1.2 and NE1m (Additional file 1: Fig. S10), we conducted pre-illumination to stabilize the baseline. After temperature equilibration and pre-illumination, we applied the following compounds in this order: NE (1 µM), DA (5 µM) followed by the α-adrenoceptor antagonist yohimbine (YO, 1 µM), and SCH (5 µM) (Fig. 3b, and Additional file 1: Fig. S5d). As we expected, DA1.2 exhibited a decrease in fluorescence to DA, but not to NE, and its response to DA was blocked by SCH treatment (Fig. 3b, c, and Additional file 1: Fig. S11a, b), confirming that the decrease in fluorescence can indeed be attributed to DA binding to DA1.2. This result suggests that the red-DA biosensor DA1.2 selectively detected DA under the presence of NE in HeLa cells. Meanwhile, NE1m showed an increase in green fluorescence upon application of NE, but not DA, and its fluorescence response recovered to its basal level following YO treatment (Fig. 3b, c, and Additional file 1: Fig. S11a, b). In summary, we demonstrated that our red-DA biosensor DA1.2 and the existing green-NE biosensor NE1m can distinguish DA and NE, respectively, in HeLa cells.

### Dual-color fluorescence imaging of DA and NE in a primary culture of rat hippocampal neurons

**Fig. 4:**
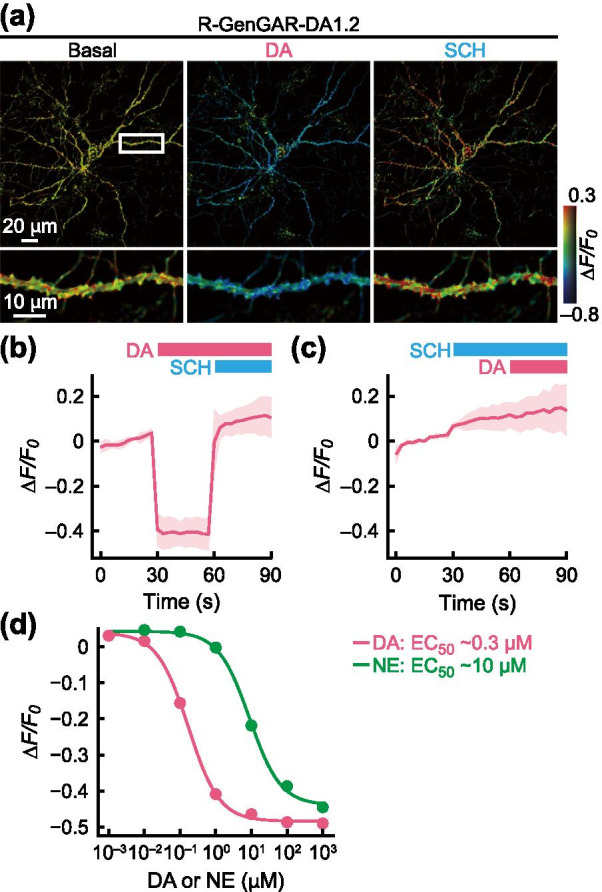
Characterization of the red-DA biosensor R-GenGAR-DA1.2 in the primary culture of rat hippocampal neurons. **a** Representative images of a primary hippocampal neuron expressing DA1.2. Top: the fluorescence change (*ΔF/F*_*0*_) before (left) and after the application of 5 μM DA (middle) followed by 5 μM SCH (right) are shown in pseudocolor intensity-modulated display mode. Bottom: magnification of dendrite marked in the top left image (white rectangle). Medium temperature was equilibrated before imaging (Additional file 1: Fig. S5b). **b** The fluorescence change (*ΔF/F*_*0*_) of DA1.2 in the primary hippocampal neurons in panel (**a**). DA (5 μM) and SCH (5 μM) were treated at the time points indicated by pink and blue bars, respectively. Mean *ΔF/F*_*0*_ values of 6 neurons from 6 experiments are shown with SD (shaded area). **c** DA1.2 was pre-treated with SCH before application of DA. Mean *ΔF/F*_*0*_ of 3 neurons from 3 experiments are shown with SD (shaded area). Medium temperature was equilibrated before imaging. **d** Temperature equilibration dose–response curves of DA (pink) and NE (green) on the primary hippocampal neurons expressing DA1.2 (Additional file 1: Fig. S4d). DA: max *ΔF/F*_*0*_ = − 0.52 ± 0.03 and EC_50_ = 0.25 ± 0.05 µM; *n* = 10 neurons from 10 experiments. NE: max *ΔF/F*_*0*_ = − 0.52 ± 0.04 and EC_50_ = 10 ± 2 µM; *n* = 7 neurons from 7 experiments. Experimental data (dots) were fitted with the Hill equation (lines). DA1.2 showed 40-fold selectivity for DA over NE in the primary culture of rat hippocampal neurons

To further test the application of DA1.2, we introduced DA1.2 into rat primary hippocampal neurons, where it was successfully expressed, and distributed in plasma membranes throughout neurons (Fig. 4a). Application of DA (5 µM) led to reduced DA1.2 red fluorescence, and this was restored to baseline following SCH treatment (5 µM) (Fig. 4a, b). Conversely, pretreatment with SCH completely suppressed the response of DA1.2 to DA (Fig. 4c), indicating that DA1.2 was successful in facilitating the visualization of DA in primary hippocampal neurons. The dose–response curve for DA and NE with temperature equilibration was obtained in primary hippocampal neurons expressing DA1.2. In this set up, DA1.2 exhibited a max *ΔF/F*_*0*_ = –0.52 ± 0.03 and an EC_50_ value of 0.25 ± 0.05 μM for DA, and a max *ΔF/F*_*0*_ = –0.52 ± 0.04 and an EC_50_ value of 10 ± 2 μM for NE (Fig. 4d) in primary hippocampal neurons. As a result, DA1.2 showed 40-fold selectivity for DA over NE in the primary culture of rat hippocampal neurons.

**Fig. 5:**
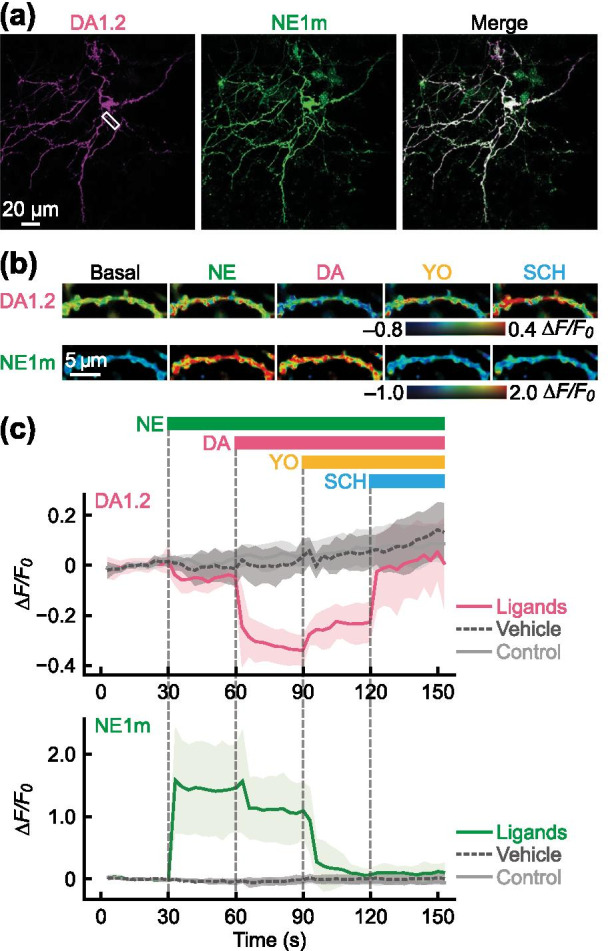
The red-DA biosensor R-GenGAR-DA1.2 combined with the green-NE biosensor GRAB_NE1m_ enables dual-color fluorescence imaging of DA and NE in a primary culture of rat hippocampal neurons. **a** Representative images of a primary hippocampal neuron co-expressing DA1.2 and NE1m. **b** Enlarged time-lapse images of DA1.2 and NE1m treated with agonists or antagonists in the pseudocolor intensity-modulated display mode from the dendritic region in the primary hippocampal neurons marked as the white boxed region in panel (**a**, left). Concentrations: DA and SCH, 5 µM; NE and YO, 1 µM. Temperature equilibration and pre-illumination were conducted before compound application (Additional file 1: Fig. S5d). **c** The fluorescence intensity change (*ΔF/F*_*0*_) of DA1.2 (top) and NE1m (bottom) in the primary hippocampal neurons co-expressing DA1.2 and NE1m. Bars show the schedule of agonist/antagonist application to both DA1.2 and NE1m. Gray vertical lines indicate time of application. Vehicle, 10 µM HCl in H_2_O or 0.1% DMSO; control, cells were only exposed to excitation light. Colored lines indicate mean *ΔF/F*_*0*_ and light-colored shaded area is the SD. Ligands, 6 neurons from 6 experiments; vehicles, 4 neurons from 4 experiments; control, 4 neurons from 1 experiment. Statistical test results are shown in Additional file 1: Fig. S11c, d

We finally performed dual-color fluorescence imaging of DA and NE in the primary culture of rat hippocampal neurons. DA1.2 and NE1m were co-expressed in the primary hippocampal neurons (Fig. 5a). After temperature equilibration and pre-illumination, we then applied the following compounds in this order: NE (1 µM), DA (5 µM) followed by YO (1 µM), and SCH (5 µM) (Additional file 1: Fig. S5d). As we observed in HeLa cells, DA, but not NE application, led to a decrease in the red fluorescence signal of DA1.2, which was restored following SCH treatment (Fig. 5b, c, and Additional file 1: Fig. S11c, d), suggesting that the red-DA biosensor DA1.2 enables selective detection of DA in the presence of NE in primary hippocampal neurons. In addition, we observed a NE-induced increase in NE1m green fluorescence, and this fluorescence response was blocked by YO treatment (Fig. 5b, c, and Additional file 1: Fig. S11c, d).

## Discussion

We independently developed genetically encoded red fluorescent DA biosensors R-GenGAR-DA1.1 and DA1.2, which respond positively and negatively to DA, respectively. Specifically, DA1.2 demonstrated a reasonable dynamic range (*ΔF/F*_*0*_ =  ~ − 40%) and DA affinity (EC_50_ =  ~ 0.9 µM) as well as high selectivity for DA (66-fold higher affinity than for NE) in HeLa cells. Taking advantage of its high selectivity, dual-color live imaging of DA in presence of NE was successfully performed using the red-DA biosensor DA1.2 combined with the existing green-NE biosensor NE1m in both HeLa cells and rat hippocampal neurons.

While we further tried to increase the selectivity of the red-DA biosensor DA1.2 by introducing mutations predicted from in silico models (Additional file 1: Fig. S6), the selectivity of DA1.2 was not raised above the already obtained level. Possibly, DA and NE are too similar in molecular structure to gain high selectivity through binding site mutations. In contrast, Gln 224 of DA1.2 which results in the 66-fold selectivity (Additional file 1: Fig. S7), is located just before cpmApple (Fig. 2a) in a region of the receptor known to move during activation [46]. Since DA1.2, relative to DA1.1, only affects NE potency (not DA potency, Additional file 1: Fig. S7c), it is likely that DA and NE preferentially stabilize distinct active states of the biosensors and that Gln 224 only impedes NE-induced activation. Distinct active states are characteristic for GPCRs signaling through multiple effectors [47], which also applies to DRD1 [48, 49]. We further compared properties of our red-DA biosensor DA1.2 with published DA biosensors, including DA affinity and selectivity for DA over NE under the same experimental condition, because calculation of EC_50_ values may be very sensitive to the experimental conditions (Additional file 1: Fig. S7). DA1.2 showed the highest selectivity to DA over NE among published DA biosensors in our experimental setup using HeLa cells (Additional file 1: Fig. S7). Utilizing the high selectivity of the red-DA biosensor DA1.2 for DA over NE, we succeeded in detecting DA and NE simultaneously in both HeLa cells and primary hippocampal neurons in vitro by dual-color imaging combined with the existing green-NE biosensor NE1m, which has high selectivity for NE over DA [34]. In addition, other advantages of a red fluorescent protein cpmApple-based DA biosensor DA1.2 are lower phototoxicity and higher tissue penetration because of its longer excitation wavelength. Furthermore, the possibility of multiplex imaging of red-DA biosensor DA1.2 combined with other colored biosensors could lead to some exciting prospects, such as investigation of different neurochemicals [50, 51], optogenetic actuators [52, 53], intracellular signaling biosensors [54, 55], calcium indicators [48, 56, 57], and voltage indicators [58]. One limitation which should be noted at this stage is the transient increase in fluorescence intensity of DA1.2 following blue-light (488 nm) illumination [59] (Additional file 1: Fig. S8c). Since random mutagenesis of the cpmApple of red-DA biosensor rGRAB_DA_ [35] was effective in reducing blue-light-induced photoactivation, introducing the mutation into cpmApple of DA1.2 would minimize the baseline fluorescence change induced by blue-light.

Despite the successful simultaneous imaging of DA and NE using red-DA biosensor DA1.2 combined with green-NE biosensor NE1m in vitro, further improvements to DA1.2 will be required for use in in vivo imaging. The main areas in which improvement are required are: [i] expanding the dynamic range and [ii] reducing baseline fluorescence changes which are dependent on temperature and excitation light. The first concern is the relatively low dynamic range of DA1.2 (*ΔF/F*_*0*_ =  ~ − 50%). Recent literature on biosensor development has shown that an increase in dynamic range can be achieved by optimization of the linker insertion site, linker length, and the amino acid sequences on the linkers and the circular-permutated fluorescent protein [35, 60, 61]. In order to expand the dynamic range of DA1.2, these strategies should be applied to DA1.2 in future work. The second concern regarding DA1.2 is changes in baseline fluorescence, which is likely due to cpmApple [45, 62, 63] and/or the entire structure of DA1.2. The temperature-dependent effect could potentially be avoided if the temperature of the animal is kept constant during in vivo imaging. Regarding the reduction of fluorescence change induced by excitation light, optimization of imaging conditions (e.g., minimal intensity of the excitation light and exposure time) requires further attention. In addition, it would be worthwhile to conduct affinity tuning to DA1.2 to increase affinity by introducing mutations to DRD1 region [33–35, 61] for in vivo use in brain regions where there is sparse dopaminergic innervation, such as the hippocampus. Furthermore, to reduce the buffering effect of DA1.2 for endogenous dopamine receptors in in vivo experiments, other improvements, for example, increasing dynamic range and brightness should help to reduce the amount of DA1.2 necessary in the brain for optimum imaging. Once these issues are overcome, an improved DA1.2 may be an extremely useful tool for the simultaneous measurements of extracellular DA and NE dynamics in the brains of freely moving animals, such as invertebrates, fish, and rodents.

Recently, there has been an increased demand for the development of tools to observe DA and NE dynamics simultaneously with high spatial and temporal resolution in vivo. For example, it was reported that pharmacological blockade of DA in D_1_/D_5_ receptors in the dorsal hippocampus prevented a memory-boosting effect induced by environmental novelty or by optogenetic activation of noradrenergic LC neurons in mice [4]. Later, Kempadoo and colleagues directly detected co-release of DA along with NE after optogenetic stimulation of LC axons in the hippocampus ex vivo using high-performance liquid chromatography [5]. These discoveries raise many questions regarding the co-release of DA and NE from LC terminals into the hippocampus in freely moving animals. For instance, what are the ranges of spatial diffusion and the precise time courses of concentration change? High spatial and temporal dual-color imaging of DA and NE dynamics in the hippocampus could give us an opportunity to answer these questions. Furthermore, when fiber photometry or two-photon microscopy is applied, the dual-color imaging will enable the measurement of DA and NE at the same spot in the brain. Because of this, the extracellular spatiotemporal dynamics of DA and NE will be comparable to each other under the same conditions.

We independently developed a novel red-DA biosensor R-GenGAR-DA1.2 which has high selectivity for DA over NE. Taking advantage of this high selectivity, we performed dual-color fluorescence live imaging using our red-DA biosensor R-GenGAR-DA1.2 and the green-NE biosensor GRAB_NE1m_ and achieved selective detection of extracellular DA even in the presence of NE at a single-neuron level in vitro. This approach will provide new insights into the high spatial and temporal dynamics of the neuromodulators DA and NE in brain areas of interest, leading to advances in our understanding of the mechanisms of interplay between DA and NE in organizing key brain functions. A better understanding of these neuromodulatory systems will have the potential to facilitate new ways of treating psychiatric disorders and neurodegenerative diseases.

## Supplementary Information

**Additional file 1:** **Figure S1.** Screening of a red fluorescent dopamine (red-DA) biosensor R-GenGAR-DA1.1. ** Figure S2.** Characterization of a red-DA biosensor R-GenGAR-DA1.1_F129A. **Figure S3.** Temperature-dependent fluorescence change and excitation-light-induced baseline fluorescence change for R-GenGAR-DA. **Figure S4.** Optimized experimental procedure for the dose-response curve. **Figure S5.** Time course of application of compounds for imaging in HeLa cells and primary hippocampal neurons. **Figure S6.** Introducing structural mutations into R-GenGAR-DA1.2.** Figure S7. **Comparison of selectivity for DA over NE between the red-DA biosensor R-GenGAR-DA and the published DA biosensors. **Figure S8. **Characterization of R-GenGAR-DA1.2. **Figure S9. **cAMP signaling in HeLa cells expressing R-GenGAR-DA1.2. **Figure S10. **Dual-color fluorescence time-lapse imaging of the red-DA biosensor R-GenGAR-DA1.2 combined with the green-NE biosensor GRAB_NE1m_ in HeLa cells with temperature equilibration (Additional file 1: Fig. S4c), but not with pre-illumination. **Figure S11.** Statistical analysis of dual-color imaging of the red-DA biosensor R-GenGAR-DA1.2 and the green-NE biosensor NE1m in HeLa cells and primary hippocampal neurons in rats. **Table S1. **Conditions for fluorescent imaging.

## Data Availability

The datasets obtained and/or analysed during the current study are available from the corresponding authors on reasonable request.

## Abbreviations

ADHD: Attention-deficit/hyperactivity disorder
CFP: Cyan fluorescent protein
cAMP: Cyclic adenosine monophosphate
DA: Dopamine
DRD1: DA receptor D1
DMSO: Dimethyl sulphoxide
EGFP: Enhanced green fluorescent protein
FB: FluoroBrite-DMEM
FRET: Fluorescence resonance energy transfer
GPCR: G protein-coupled receptor
LC: Locus coeruleus
mTFP: Monomeric teal fluorescent protein
NE: Norepinephrine
PCR: Polymerase chain reaction
SCH: SCH 23390
YFP: Yellow fluorescent protein
YO: Yohimbine
