# Supplementary material for: A novel red fluorescence dopamine biosensor selectively detects dopamine in the presence of norepinephrine in vitro
Source: Mol Brain. 2021 Dec 6;14:173. doi: 10.1186/s13041-021-00882-8 (PMC8647500; doi:10.1186/s13041-021-00882-8)
Supplement: Supplementary file 1 — Additional file 1: Figure S1. Screening of a red fluorescent dopamine (red-DA) biosensor R-GenGAR-DA1.1. Figure S2. Characterization of a red-DA biosensor R-GenGAR-DA1.1_F129A. Figure S3. Temperature-dependent fluorescence change and excitation-light-induced baseline fluorescence change for R-GenGAR-DA. Figure S4. Optimized experimental procedure for the dose-response curve. Figure S5. Time course of application of compounds for imaging in HeLa cells and primary hippocampal neurons. Figure S6. Introducing structural mutations into R-GenGAR-DA1.2. Figure S7. Comparison of selectivity for DA over NE between the red-DA biosensor R-GenGAR-DA and the published DA biosensors. Figure S8. Characterization of R-GenGAR-DA1.2. Figure S9. cAMP signaling in HeLa cells expressing R-GenGAR-DA1.2. Figure S10. Dual-color fluorescence time-lapse imaging of the red-DA biosensor R-GenGAR-DA1.2 combined with the green-NE biosensor GRABNE1m in HeLa cells with temperature equilibration (Additional file 1: Fig. S4c), but not with pre-illumination. Figure S11. Statistical analysis of dual-color imaging of the red-DA biosensor R-GenGAR-DA1.2 and the green-NE biosensor NE1m in HeLa cells and primary hippocampal neurons in rats. Table S1. Conditions for fluorescent imaging. [file 13041_2021_882_MOESM1_ESM.docx]

**Additional file 1**

**Additional file 1: Figure S1.** Screening of a red fluorescent dopamine (red-DA) biosensor R-GenGAR-DA1.1. **a** Time-lapse imaging of R-GenGAR-DA1.0_1. Mean *ΔF/F_0_* of 10 cells is shown with SD (shaded area). Dopamine (DA, 10 μM) was applied at the time point shown by the pink bar. **b** Time-lapse imaging of DA1.0_310 and DA1.0_430. Mean *ΔF/F_0_* of 10 cells are shown with SD (shaded area). DA (10 μM) and SCH 23390 (SCH, 10 μM) were treated at the indicated time points shown by pink and blue bars, respectively. **c** The amino acid sequence of linker sequences for DA1.0_1, DA1.1 (DA1.0_76), DA1.0_310, and DA1.0_430, which were obtained from 1st screening.

**Additional file 1: Figure S2.** Characterization of a red-DA biosensor R-GenGAR-DA1.1_F129A. **a** Schematic illustration of DA1.1_F129A. Phe 129, located in DRD1 intracellular loop 2, mutated to alanine (F129A). **b** Time-lapse imaging of DA1.1_F129A. DA (10 μM) and SCH (10 μM) were treated at the indicated time points. Mean *ΔF/F_0_* of 20 cells from 2 independent experiments are shown with SD (shaded area).

**Additional file 1: Figure S3.** Temperature-dependent fluorescence change and excitation-light-induced baseline fluorescence change for R-GenGAR-DA. **a–d** Representative images of HeLa cells expressing DA1.1 (**a**), and DA1.2 (**c**) shown in the pseudocolor intensity-modulated display mode in various incubation temperatures. Regression curve of the fluorescence intensity change (*ΔF/F_0_*) of DA1.1 (**b**), and DA1.2 (**d**) and incubation temperature. The fluorescence intensity of DA1.1 and DA1.2 was shown to change in a temperature-dependent manner of the culture medium maintained by 5% CO_2_ and HEPES. Negative correlation between fluorescent intensity and temperature in DA1.1 (r = –0.96, 20 cells in 2 experiments) and DA1.2 (r = –0.93, 30 cells in 3 experiments) were observed by Pearson product moment correlation coefficient. **e** Excitation-light-induced baseline fluorescence change in HeLa cells expressing DA1.2 under the indicated excitation light conditions (excitation light wavelength, excitation light power, and exposure time; Additional file 1: Table S1) through a 20× objective lens. Excitation and fluorescence measurements were recorded every 3 s. Increase in basal red fluorescence of DA1.2 was most pronounced when a larger power was used for the excitation light (e.g. 561 nm, 100%, 1000 ms, and both 488 nm and 561 nm, 100%, 1000 ms). The colored-lines represent the average values with the SD of them (shaded area) (*n* = 10 cells in each case). Differences amongst area under the curves from 4 exposure conditions were tested as follows. Normality assumption was judged from Shapiro-Wilk test and Q-Q plot. Variances among conditions was assumed equal following Bartlett test (*p* = 0.696). One-way ANOVA was performed (*F*_3,36_ = 110, *p* < 0.01). As a post-hoc analysis, Tukey-Kramer was used for multiple comparisons (n.s., nonsignificant; **p* < 0.05; ***p* < 0.01). **f** Excitation-light-induced baseline fluorescence change in primary hippocampal neuron expressing DA1.2. Light illumination protocol as follows: 1-s exposure at 561 nm (excitation light power, 10%) followed by 1-s exposure at 488 nm (excitation light power 5%); excitation and fluorescence measurement was performed at every 3 s through a 60× Oil objective lens. Mean intensities from first 30 s of each phase were used as *F_0_*. The mean *ΔF/F_0_* values in first 150-s imaging gradually increased, while those in the second 150-s imaging was relatively constant and stable. The temperature of the culture medium was constant during time-lapse imaging. Mean *ΔF/F_0_* values of first (blue) and second (orange) 150-s imaging are shown with the SD of them (shaded area) (*n* = 4 neurons). Difference between area under the curve of first and second imaging was tested by a two-tailed paired *t*-test (***p* = 0.007).

**Additional file 1: Figure S4.** Optimized experimental procedure for the dose-response curve. **a** Dishes for imaging of HeLa cells and primary hippocampal neurons. **b** Application of compounds for imaging. The compounds, mixed with the 0.5 ml imaging buffer from the well of interest, was applied at the time of imaging. **c** Temperature equilibration for imaging. Imaging buffer (0.5 ml out of 1 ml for HeLa cells, and 2 ml for the neurons) from the well of interest transferred to the empty 1.5-ml microcentrifuge tube and returned to the same well and repeated five times. This procedure gradually equilibrated the temperature of the imaging buffer to room temperature and effected the basal fluorescence level of DA1.1 and DA1.2 stable. **d** Procedure for making the dose-response curve. Top: the time course of ligand application and imaging shown by the arrow after temperature equilibration. Application of the diluted ligand, imaged sequentially. Bottom: representative images of DA1.2, negatively responding to DA in a dose-dependent manner. **e** Quantification of snapshots in the HeLa cells expressing DA1.2 in the dose-response curve for DA without (left) or with (right) temperature equilibration. Temperature equilibration effected to stabilize the basal level *ΔF/F_0_* values of DA1.2. (Left, *n* = 4 cells; right, *n* = 3 cells). **f** Confirmation of basal stability of DA1.1, and DA1.2. HeLa cells expressing DA1.1 (left) or DA1.2 (right) were treated with 7 trials of vehicle application after temperature equilibration, showing no change in the mean *ΔF/F_0_* values with the SEM of them (*n* = 3 experiments in each case). The procedure is the same as a panel (**d**).

**Additional file 1: Figure S5.** Time course of application of compounds for imaging in HeLa cells and primary hippocampal neurons. **a** Compound application for time-lapse imaging without temperature equilibration. Compound or vehicle applied with imaging buffer from the well of interest shown by the arrow. Cells were imaged with the appropriate time exposure (Additional file 1: Table S1) acquired every 3 s for a duration of 90 s. **b** Compound application with temperature equilibration for time-lapse imaging. Before compound application, temperature equilibration conducted as shown in Additional file 1: Fig. S4c. Cells were imaged with the appropriate time exposure (Additional file 1: Table S1) acquired every 3 s for a duration of 90 s. **c** Compound application for checking pharmacological selectivity of DA1.2. Cells were imaged with the appropriate time exposure (Additional file 1: Table S1) acquired every 3 s for a duration of 60 s. Averaged *ΔF/F_0_* during 30-60 s of each compound was shown in Fig. 2e. **d** Compound application for dual-color imaging. After temperature equilibration, we conducted pre-illumination, i.e. dual-color light illumination (1-s exposure of 561-nm followed by 1-s exposure of 488-nm light illumination) every 3 s for a duration of 150 s, to reduce the effect of excitation-light-induced baseline fluorescence change. Cells were then dual-color imaged (561-nm followed by 488-nm light illumination) with the appropriate time exposure (Additional file 1: Table S1) acquired every 3 s for a duration of 150 s.

**Additional file 1: Figure S6.** Introducing structural mutations into R-GenGAR-DA1.2. **a** Prediction of the residues responsible for the selectivity between DA and NE from structural models of the DRD1 (dark blue cartoon and white sticks) with either DA (left, salmon sticks) or NE (right, green sticks) in the binding site. The amino acids close to the additional hydroxy of NE (i.e. Ser 107, Val 317 and Trp 321) may be utilized to affect the preference for binding of DA over NE, e.g. by mutation of hydrogen bonding (yellow dotted lines) amino acids with hydrophobic ones. **b** Candidates of structural mutation. **c** Mean *ΔF/F_0_* (20 cells from 2 experiments in each case, except DA1.2_V317M, where 10 cells from 1 experiment were used) are shown with the SD of them (shaded area). DA (10 μM) and SCH (10 μM) were treated at the indicated time points shown by pink and blue bars, respectively. **d** Box plot shows averaged *ΔF/F_0_* during DA application (30-s duration) of each mutant. **e** Dose-response curves with temperature equilibration (Additional file 1: Fig. S4d) of DA (pink) and NE (green) in HeLa cells expressing DA1.2_V317I, DA1.2_V317M, and DA1.2_W321H. DA1.2_V317I: DA: max *ΔF/F_0_* = 0.49 ± 0.01 and EC_50_ = 1.10 ± 0.24 µM; NE: max *ΔF/F_0_* = 0.47 ± 0.03 and EC_50_ = 55 ± 14 µM; 50-fold selectivity for DA over NE (DA and NE, *n* = 3 experiments in both cases). DA1.2_V317M: DA: max *ΔF/F_0_* = 0.43 ± 0.02 and EC_50_ = 0.66 ± 0.11 µM; NE: max *ΔF/F_0_* = 0.42 ± 0.02 and EC_50_ = 19.0 ± 4.1 µM; 29-fold selectivity for DA over NE (DA and NE, *n* = 3 experiments in both cases). DA1.2_W321H: DA: max *ΔF/F_0_* = 0.55 ± 0.06 and EC_50_ = 12.0 ± 7.4 µM; NE: max *ΔF/F_0_* = 0.62 ± 0.07 and EC_50_ = 111 ± 12 µM; 9-fold selectivity for DA over NE (DA and NE, *n* = 3 experiments in both cases). Experimental data (dots) were fitted with the Hill equation (lines).

**Additional file 1: Figure S7.** Comparison of selectivity for DA over NE between the red-DA biosensor R-GenGAR-DA and the published DA biosensors. **a** Dose-response curve for DA (pink) and NE (green) in HeLa cells expressing green-DA biosensors (dLight1.1, dLight1.2 and dLight1.3a). dLight1.1: DA: max *ΔF/F_0_* = 0.95 ± 0.05 and EC_50_ = 0.71 ± 0.08 µM; NE: max *ΔF/F_0_* = 0.78 ± 0.11 and EC_50_ = 12 ± 1 µM (DA and NE, *n* = 4 independent experiments in both cases). dLight1.2: DA: max *ΔF/F_0_* = 4.2 ± 0.2 and EC_50_ = 2.3 ± 0.3 µM; NE: max *ΔF/F_0_* = 2.8 ± 0.3 and EC_50_ = 73 ± 5 µM (DA and NE, *n* = 4 independent experiments in both cases). dLight1.3a: DA: max *ΔF/F_0_* = 4.9 ± 0.5 and EC_50_ = 3.8 ± 0.3 µM; NE: max *ΔF/F_0_* = 3.9 ± 0.4 and EC_50_ = 74 ± 5 µM (DA and NE, *n* = 4 independent experiments in both cases). **b** Dose-response curve for DA (pink) and NE (green) in HeLa cells expressing red-DA biosensors (rGRAB_DA1h_, rGRAB_DA1m_, and RdLight1). rGRAB_DA1h_: DA: max *ΔF/F_0_* = 1.3 ± 0.1 and EC_50_ = 0.06 ± 0.02 µM; NE: max *ΔF/F_0_* = 1.1 ± 0.04 and EC_50_ = 0.10 ± 0.01 µM (DA and NE, *n* = 3 independent experiments in both cases). rGRAB_DA1m_: DA: max *ΔF/F_0_* = 1.4 ± 0.1 and EC_50_ = 0.21 ± 0.03 µM; NE: max *ΔF/F_0_* = 1.2 ± 0.1 and EC_50_ = 1.8 ± 0.2 µM (DA and NE, *n* = 3 independent experiments in both cases). RdLight1: DA: max *ΔF/F_0_* = 3.1 ± 0.03 and EC_50_ = 1.4 ± 0.4 µM; NE: max *ΔF/F_0_* = 2.4 ± 0.4 and EC_50_ = 36 ± 2 µM (DA and NE, *n* = 3 independent experiments in both cases). **c** Summarized affinity for DA and NE, and selectivity for DA over NE of R-GenGAR-DA1.1, R-GenGAR-DA1.2, dLight1.1, dLigh1.2, dLight1.3a, rGRAB_DA1h_, rGRAB_DA1m_, and RdLight1. Selectivity was calculated using EC_50_ of NE relative to EC_50_ of DA.

**Additional file 1: Figure S8.** Characterization of R-GenGAR-DA1.2. **a** Normalized excitation spectrum (orange) and emission spectrum (red) of DA1.2. The fluorescence intensity was normalized by setting the maximum value as 100% in each emission and excitation spectrum. The peak absorption was 566 nm, and the emission spectrum was 595 nm. **b** Averaged relative brightness of red-DA biosensors, DA1.2, RdLight1, rGRAB_DA1m_, and rGRAB_DA1h_ are shown before (gray) and after (pink) DA stimulation (10 µM) with SEM (*n* = 3 independent experiments in both cases). The fluorescence intensity was normalized by setting the DA1.2 without DA stimulation as 1. **c** Blue-light induced photoactivation of DA1.2. The fluorescence intensity was normalized by setting the initial time point as 1. The mean of normalized fluorescence intensity is plotted as a function of time using the SD (*n* = 17 cells). **d** Signal-to-noise ratio of DA1.1 and DA1.2 in HeLa cells. The values were calculated as the response divided by the standard deviation of the fluorescence fluctuation.

**Additional file 1: Figure S9.** cAMP signaling in HeLa cells expressing R-GenGAR-DA1.2. **a** Schematic illustration of the cAMP biosensor containing CFP, Exchange protein directly activated by cAMP 1 (EPAC 1), and YFP. In the absence of cAMP, the cAMP sensor is the closed-form and high FRET state (left). Once cAMP binds, the cAMP sensor undergoes a structural change to become the open-form and low FRET state (right). **b** Representative images of DRD1 (left), DA1.2 (middle), and control (right, empty vector), which were co-expressing the cAMP biosensor, before (top) and after (bottom) application of DA shown in the pseudocolor intensity-modulated display mode. **c** Time-lapse imaging of cAMP level (CFP/FRET) in HeLa cells expressing DRD1 (blue), DA1.2 (pink), and control (gray). DA (1 µM) was treated at the time points shown by the pink bar. Cells were imaged with the appropriate time exposure (Additional file 1: Table S1) acquired every 1 min for a duration of 30 min. Mean CFP/FRET of 20 cells in 2 experiments is shown with the SD of them (shaded area).

**Additional file 1: Figure S10.** Dual-color fluorescence time-lapse imaging of the red-DA biosensor R-GenGAR-DA1.2 combined with the green-NE biosensor GRAB_NE1m_ in HeLa cells with temperature equilibration (Additional file 1: Fig. S4c), but not with pre-illumination. Bars show the schedule of agonist/antagonist application to both DA1.2 and NE1m. Gray vertical lines indicate time of application. Concentrations: DA and SCH, 5 µM; NE and YO, 1 µM. The fluorescence intensity change (*ΔF/F_0_*) of DA1.2 (top) and NE1m (bottom) in HeLa cells co-expressing DA1.2 and NE1m. Vehicle, 10 µM HCl in H_2_O or 0.1% DMSO; control, cells were only exposed to excitation light. Mean *ΔF/F_0_* values of 30-40 cells from 3-4 experiments are shown with SD (shaded areas).

**Additional file 1: Figure S11.** Statistical analysis of dual-color imaging of the red-DA biosensor R-GenGAR-DA1.2 and the green-NE biosensor NE1m in HeLa cells and primary hippocampal neurons in rats. **a**, **c** Quantification of time-lapse imaging from Fig. 3c and Fig. 5c. Each *ΔF/F_0_* value for a given compound was normalized by the subtraction of averaged vehicle values along the time course. Data are represented using box plots for each consecutive step in the experiment. Each box represents of the final 15 s (5 time points) of each 30 s condition, which occurs immediately prior to the application of each successive compound. The order of boxes from left to right reflects the time course. (HeLa cells, *n* = 30 cells; neuron, *n* = 6 cells). **b**, **d** Statistical results of panels (**a**) and (**c**), respectively. There were significant differences between compounds analyzed by Friedman test in HeLa cells (DA1.2, *p* < 0.001; NE1m, *p* < 0.001) and in hippocampal primary neurons (DA1.2, *p* < 0.001; NE1m, *p* < 0.001). Conover-Iman test with the Bonferroni-Holm correction for multiple testing, as a post-hoc analysis, *p* values are shown in the table. n.s., nonsignificant.

**Additional file 1: Table S1.** Conditions for fluorescent imaging.

| **Figure** | **Sensor** | **Cell-type** | **Microscopy** | **Filters** | **Exposure time:**  **laser power** |
| --- | --- | --- | --- | --- | --- |
| Fig. 1b | DA1.0 | HeLa | IXM-XLS  10× (NA = 0.30)  20× (NA = 0.75) | Ex: 562/40  Dichroic:  350-585/601-950 (T)  Em: 624/40 | 1000 ms  (Lumen cor 100/255) |
| Fig. 1d  Fig. S1 a, b  Fig. S2b | DA1.0  DA1.1 | HeLa | IX83  20× (NA = 0.75)  20× (NA = 0.80) | Ex: 561 nm  Dichroic:  DM405/488/561  Em: 580–654 nm | 500 ms  (Lumen cor 100/255) |
| Fig. 1e  Fig. 2b–e  Fig. S3a–d  Fig. S4d  Fig. S6c–e | DA1.1  DA1.2 | HeLa | IX83 with CSU-W1  20× (NA = 0.75)  20× (NA = 0.80) | Ex: 561 nm  Dichroic:  DM405/488/561  Em: 580–654 nm | 200 ms (ND 100 %) |
| Fig. 2f | DA1.2 | HeLa | IX83 with CSU-W1  60× (NA = 1.42) | Ex: 561 nm  Dichroic:  DM405/488/561  Em: 580–654 nm | 20 ms (ND 5 %) |
| Fig. S3e | DA1.2 | HeLa | IX83 with CSU-W1  20× (NA = 0.75)  20× (NA = 0.80) | Ex1: 561 nm  Ex2: 488 nm  Dichroic:  DM405/488/561  Em1: 580–654 nm  Em2: 500-550 nm | Ex1: 1000 ms (ND 100%)  Ex1: 1000 ms (ND 50%)  Ex1, Ex2: 1000 ms (ND 100%)  Ex1, Ex2: 200 ms (ND 50%) |
| Fig. 3  Fig. S10 | DA1.2  NE1m | HeLa | IX83 with CSU-W1  20× (NA = 0.75)  20× (NA = 0.80) | Ex1: 561 nm  Ex2: 488 nm  Dichroic:  DM405/488/561  Em1: 580–654 nm  Em2: 500-550 nm | Ex1: 200 ms (ND 100%)  Ex2: 200 ms (ND 100%) |
| Fig. 4 | DA1.2 | Neuron | IX83 with CSU-W1  60× Oil (NA = 1.35)  60× Oil (NA = 1.42) | Ex: 561 nm  Dichroic:  DM405/488/561  Em: 580–654 nm | 1000 ms (ND 10 %) |
| Fig. 5  Fig. S3f | DA1.2  NE1m | Neuron | IX83 with CSU-W1  60× Oil (NA = 1.35)  60× Oil (NA = 1.42) | Ex1: 561 nm  Ex2: 488 nm  Dichroic:  DM405/488/561  Em1: 580–654 nm  Em2: 500-550 nm | Ex1: 1000 ms (ND 10 %)  Ex2: 1000 ms (ND 5%) |
| Fig. S7a | dLigh1.1  dLight1.2  dLight1.3a | HeLa | IX83  20× (NA = 0.75) | Ex: 488 nm  Dichroic:  DM405/488/561  Em: DM405/488/561 | 500 ms  (Lumen cor 20/255) |
| Fig. S7b  Fig. S8d | rGRAB_DA1h_  rGRAB_DA1m_  RdLight1  DA1.1 DA1.2 | HeLa | IX83 with CSU-W1  20× (NA = 0.80) | Ex: 561 nm  Dichroic:  DM405/488/561  Em: 580–654 nm | 200 ms (ND 100 %) |
| Fig. S8a | DA1.2 | HeLa | Leica SP8 Falcon  20× (NA = 0.75) | Ex1: 540 nm  Em1: 550-697 nm (10 nm window)  Ex2: 470-614 nm  Em2: 550-780 nm | Ex1: 10%- |
| Fig. S8b | DA1.2  RdLight1  rGRAB_DA1m_  rGRAB_DA1h_ | HeLa | IX83 with CSU-W1  20× (NA = 0.8) | Ex1: 561 nm  Ex2: 488 nm  Dichroic:  DM405/488/561  Em1: 580–654 nm  Em2: 500-550 nm | Ex1: 500 ms (ND 25 %)  Ex2: 100 ms (ND 1%) |
| Fig. S8c | DA1.2 | HeLa | IX83 with CSU-W1  60× Oil (NA = 1.42) | Ex1: 561 nm  Ex2: 488 nm  Dichroic:  DM405/488/561  Em1: 580–654 nm  Em2: 500-550 nm | Ex1: 10 ms (ND 25 % ~ 7.4 mW/cm^2^)  Ex2: 50 ms (ND 100% ~ 23 mW/cm^2^) |
| Fig. S9 | CFP-EPAC1  -YFP | HeLa | IX83 with CSU-W1  20× (NA = 0.75) | Ex: 440 nm  Dichroic:  DM445/514/640  Em (CFP): 465-500 nm  Em (FRET): 500-550 nm | 500 ms (ND 25%) for CFP  500 ms (ND 25%) for FRET |
